# Influence of the watch and wait strategy on clinical outcomes of patients with follicular lymphoma in the rituximab era

**DOI:** 10.1007/s00277-016-2800-1

**Published:** 2016-09-26

**Authors:** Sayako Yuda, Dai Maruyama, Akiko Miyagi Maeshima, Shinichi Makita, Hideaki Kitahara, Ken-ichi Miyamoto, Suguru Fukuhara, Wataru Munakata, Tatsuya Suzuki, Yukio Kobayashi, Kinuko Tajima, Hirokazu Taniguchi, Kensei Tobinai

**Affiliations:** 1 Department of Hematology, National Cancer Center Hospital, 5-1-1 Tsukiji, Chuo-ku, Tokyo, 104-0045 Japan; 2 Department of Pathology and Clinical Laboratory, National Cancer Center Hospital, Tokyo, Japan; 3 Course of Advanced Clinical Research of Cancer, Juntendo University Graduate School of Medicine, Bunkyō, Japan

**Keywords:** Follicular lymphoma, Watch and wait, Time to treatment failure, Rituximab

## Abstract

We analyzed the effects of the initial approach to patients with follicular lymphoma (FL) on outcomes in order to investigate whether the watch and wait (WW) strategy is still an acceptable approach in the rituximab era. We retrospectively analyzed 348 patients who were initially diagnosed with FL between 2000 and 2012. We compared the clinical outcomes of the WW cohort and immediate treatment cohort. Among 348 patients (median age of 57 years, range: 19–85), 101 were initially managed with WW and 247 were immediately treated. The median follow-up duration was 75 months (range: 7–169). The estimated median time to treatment failure (TTF) in the treatment following WW cohort and immediate treatment cohort were 92 months (95 % CI, 60.1–NA) and 77 months (95 % CI, 65.1–107.6), respectively, which were not significantly different (*P* = 0.272) . In a multivariate analysis, clinical stage was identified as a predictive factor of TTF (HR 1.19, 95 % CI, 1.03–1.38, *P* < 0.05). Neither overall survival rate nor cumulative risk of transformation between the WW cohort and immediate treatment cohort was significant. The results of the present study suggested that the WW strategy is still an acceptable approach for selected FL patients in the rituximab era.

## Introduction

In the pre-rituximab era, several studies revealed that the watch and wait (WW) strategy was not associated with the outcomes of patients with follicular lymphoma (FL) regardless of their tumor burden [1–7]. These findings confirmed that WW may be one of the standard approaches for patients with asymptomatic FL. However, it is currently being debated whether WW is still acceptable in the rituximab era.

In recent years, the role of WW has been examined in more detail in patients with FL. Two retrospective studies concluded that the WW strategy is still an acceptable approach for selected patients [8, 9]. Another two prospective studies reported that the outcomes of a cohort managed with WW were poorer in those treated with rituximab-containing therapy [10, 11]. Therefore, in the rituximab era, there are no longer adequate grounds for the immediate treatment of patients with FL. We herein report the results of our retrospective study on the effects of the initial approach to patients with FL on outcomes.

## Patients and methods

Patients who were newly diagnosed with FL grades 1 to 3a in accordance with the World Health Organization classification [12] at the National Cancer Center Hospital between 2000 and 2012 were included irrespective of age, Ann Arbor stage, Eastern Cooperative Oncology Group performance status, symptoms, tumor burden, Follicular Lymphoma International Prognostic Index 2 (FLIPI2) [13], and time to start the initial treatment. In the present study, a high tumor burden (HTB) was defined as cases with at least one of the following items: the largest mass of more than 7 cm, more than three nodal sites with a diameter >3 cm, significant serous effusion, organ compression, and symptomatic splenomegaly [4]. Exclusion criteria were the presence of a histological transforming component such as diffuse large B cell lymphoma (DLBCL) at the time of the initial diagnosis or patients who were enrolled in new agent clinical trials. The decision on when to start the initial treatment was made by the responsible physicians. Patients were managed in accordance with good practice rules. Clinical data were collected from our medical records, including patient baseline characteristics, initial approaches, responses after the treatment, and reasons for starting the treatment following WW. We evaluated overall survival (OS), time to treatment failure (TTF), and the cumulative risk of transformation. Judgments on transformation were made pathologically and/or clinically. Clinical transformation was considered, for example, when the patient exhibited rapid elevations in lactate dehydrogenase, rapid growth of the tumor, B symptoms, or hypercalcemia.

We compared the outcomes of patients who were treated following WW with those who were immediately treated. The WW cohort was defined as patients who did not received the initial treatment in the first 3 months of the diagnosis. In the WW cohort, the clinical features of patients who needed to be treated after observations were also analyzed.

TTF was defined as the time from the initial diagnosis to progression or death after the first treatment regardless of the initial approach. Patients who continued WW during the follow-up were not included as subjects for TTF. OS was defined as the time from the initial diagnosis to death by any cause and was analyzed using the Kaplan-Meier method. In the analysis of OS, transformation was regarded as a time-dependent covariate. Cox’s proportional hazards model was used to assess relationships between clinical variables and treatment failure or death. The cumulative incidence of transformation was determined by using death as a competing risk. The cumulative risk of transformation was estimated using the Gray method. The relationship between clinical variables and transformation was analyzed using the Fine-Gray proportional hazards models [14]. Fisher’s exact test was used to compare two categorical variables. In this study, two-sided *p* values less than 0.05 were considered significant. All analyses were performed with EZR (Easy R) version 1.32 (Saitama Medical Center, Jichi Medical University), which is a graphical user interface for R (The R Foundation for Statistical Computing, version 3.2.2) [15].

This study was conducted according to the provisions of the Declaration of Helsinki. The Institutional Review Board of the National Cancer Center approved this study protocol.

## Results

### Patient characteristics

Between January 2000 and December 2012, 348 patients, with a median age of 57 years (range: 19–85 years), were newly diagnosed with FL grades 1 to 3a without the components of DLBCL in our institution. According to the physician’s discretion, 101 patients were initially managed with WW and 247 were immediately treated.

The baseline characteristics of patients are shown in Table 1. No patients with B symptoms were observed in the WW cohort. Clinical physicians preferred to immediately treat patients with histological grade 2 or 3a, FLIPI2 high, performance status 1 or higher, or HTB.

**Table 1:** Patient characteristics at the initial diagnosis

	WW cohort (*N* = 101)		Immediate treatment cohort (*N* = 247)		*P*
Characteristics	No.	%	No.	%	
Age (years)					
Median	59		57		0.07
Range	35–85		19–85		
>60	45	45 %	84	34 %	
Male	52	51 %	112	45 %	0.34
Performance status					
0	97	96 %	193	78 %	<0.001
1	4	4 %	53	21 %	
2	0	0 %	1	0 %	
Histological grade					
1	55	54 %	90	36 %	0.004
2	35	35 %	102	41 %	
3a	11	11 %	55	22 %	
Ann Arbor stage					
1	24	24 %	54	22 %	0.100
2	18	18 %	22	9 %	
3	18	18 %	48	19 %	
4	41	41 %	123	50 %	
Bone marrow involvement	32	32 %	96	39 %	0.22
B symptoms	0	0 %	12	5 %	0.02
FLIPI2					
Low	58	57 %	120	49 %	0.034
Intermediate	20	20 %	51	21 %	
High	8	8 %	46	19 %	
Unknown	15	15 %	30	12 %	
High tumor burden	20	20 %	130	53 %	<0.001

*WW* watch and wait, *FLIPI* Follicular Lymphoma International Prognostic Index

### Treatment following WW

In the WW cohort, 45 patients (45 %) received an anti-lymphoma treatment after a median WW duration of 16 months (range: 3–122 months). The reasons for starting this treatment after WW are listed in Table 2, with the progression of tumors being the most common reason (58 %). Sixteen patients advanced to HTB from a low tumor burden (LTB) during WW. Treatments following WW included rituximab plus chemotherapy in 27 patients (60 %), rituximab monotherapy in 11 (24 %), radiotherapy alone in 4 (9%), and chemotherapy alone in 3 (7%) (Table 3). No patients received rituximab maintenance therapy during the study period.

**Table 2:** Reasons for starting the treatment after WW (*N* = 45)

Reason	No.	%
Progression of tumors	26	58
Development of symptoms	9	20
Organ compression	4	9
Patient request	3	7
Transformation	2	4
Other	1	2

*WW* watch and wait

**Table 3:** Initial treatments

	Treatment following WW (*N* = 45)		Immediate treatment (*N* = 247)	
Treatment	No.	%	No.	%
Rituximab monotherapy	11	24	20	8
Rituximab + chemotherapy	27	60	169	68
Chemotherapy	3	7	7	3
Radiation therapy only	4	9	35	14
Radiation + chemotherapy ± rituximab	0	0	16	6

*WW* watch and wait

### Response and TTF

**Fig. 1:**
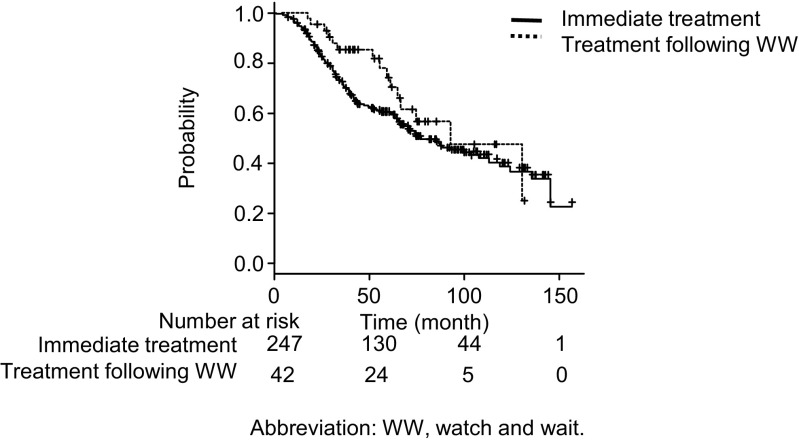
TTF of the treatment following WW cohort and immediate treatment cohort

By the end of the follow-up, 289 patients including 42 in the WW cohort and 247 in the immediate cohort had completed the initial treatment. Responses to the initial treatment were as follows: 228 patients achieved a complete response, 54 partial responses, two stable diseases, and five progressive diseases. Among the patients who received the initial treatment during the follow-up, 136 were regarded as treatment failures after the initial treatment: 15 out of 42 (36%) in the WW cohort and 121 out of 247 (49 %) in the immediate treatment cohort. The estimated median TTF were 92 months (95 % CI, 60.1–NA) in the WW cohort and 77 months (95 % CI, 65.1–107.6) in the immediate treatment cohort, which were not significantly different (*P* = 0.272) (Fig. 1). In a multivariate analysis, clinical stage 3 or 4 was identified as a predictive factor for TTF (HR 1.19, 95 % CI, 1.03–1.38, *P* < 0.05).

### OS

**Fig. 2:**
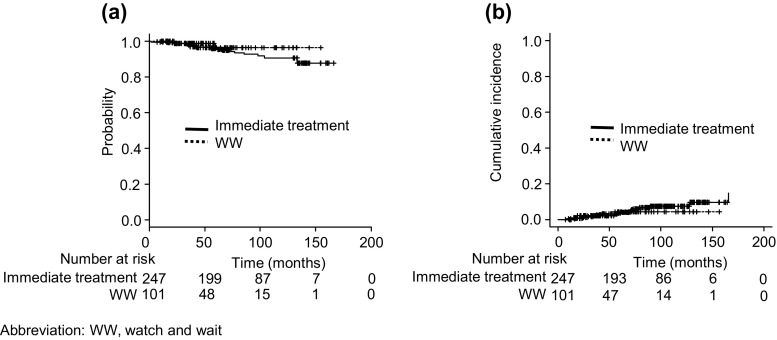
**a** OS of the WW cohort and immediate treatment cohort. **b** Cumulative incidence of transformation of the WW cohort and immediate treatment cohort

Nineteen (6 %) patients died with a median follow-up of 75 months (range: 7–169). The causes of their deaths included the progression of FL in 8 patients: 5 had no evidence of transformation, 2 clinically transformed FL, and 1 pathologically transformed FL; secondary malignancies in 8: 2 with acute lymphoblastic leukemia, 2 acute myeloid leukemia, 1 primary unknown small cell carcinoma, 1 lung cancer, and 1 esophageal cancer; idiopathic pulmonary fibrosis in 1; acute heart failure of unknown etiology in 1; and unknown in 1. The difference in OS rates between the WW cohort and the immediate treatment cohort was not significant (*P* = 0.294) (Fig. 2a). Clinical stage (HR 2.05, 95 % CI, 1.12–3.75, *P* < 0.05), age >60 years (HR 3.87, 95 % CI, 1.52–9.85, *P* < 0.05), and transformation (as a time-dependent covariate) (HR 6.15, 95 % CI, 1.64–23.02, *P* < 0.05) were identified as risk factors for death in the multivariate analysis.

### Incidence of transformation

Nineteen patients (6 %) exhibited transformation: 3 in the WW cohort and 16 in the immediate treatment cohort. Only three patients were proven to have pathologically transformed FL while the remaining patients showed clinical transformation. The median time to events was 54 months (range: 9–166). The cumulative risk of transformation in the two cohorts was similar (*P* = 0.64) (Fig. 2b). The cumulative incidence rates of transformation at 5 and 10 years were 4.4 % (95 % CI, 1.0–11.8) and 4.4 % (95 % CI, 1.0–11.8) in the WW cohort and 3.6 % (95 % CI, 1.7–6.7) and 7.6 % (95 % CI, 4.2–12.1) in the immediate treatment cohort, respectively. In the multivariate analysis, none of the baseline characteristics or initial approaches was significant for the risk of transformation.

### Subgroup analysis by tumor burden

We performed subgroup analyses of TTF, OS, and transformation rates in all patients according to their tumor burden. The WW cohort had 20 patients with HTB and 81 with LTB, while the immediate treatment cohort had 130 with HTB and 117 with LTB. In both subgroups, no significant differences were observed in the TTF, OS, or transformation rates between the two cohorts.

## Discussion

This was a retrospective analysis that focused on the influence of the WW strategy for patients with newly diagnosed FL in the rituximab era. We analyzed the clinical outcomes of the whole population of FL in our institution in order to identify patients manageable with WW in the rituximab era.

In the present study, TTF, which was defined as the time from the initial diagnosis to progression or death after the first treatment, was considered to be one of the reasonable endpoints, while some research on FL selected progression-free survival (PFS) for the primary endpoint, the time from start of the initial treatment, or WW to disease progression or death. The difference between TTF in our study and PFS was whether WW is regarded as one of the initial treatments. Since it is not rare for FL to regrow repeatedly after responding to previous treatments, it is necessary to use TTF in order to verify whether the WW strategy has the potential to successfully postpone the initial treatment of FL.

In the present study, the estimated TTF in patients treated following WW was 92 months, which appears to be better than those reported recently. Solal-Cligny et al. retrospectively investigated 107 patients with LTB in the F2-study database who were initially managed with WW. The 4-year freedom from treatment rate was 79 %, which was not inferior to that of patients with LTB who were initially treated with a rituximab-based regimen [8]. In a randomized phase III trial conducted by Ardeshna et al., 379 patients with LTB were randomly assigned to the WW arm, rituximab induction arm, or rituximab induction followed by a rituximab maintenance arm. They reported that the time to the start of the new treatment in the WW arm was significantly shorter than the time to the next treatment in the other two arms [10]. Although only patients with no symptoms with LTB were evaluated in their phase III trial, we analyzed the clinical data of patients with FL irrespective of their symptoms and tumor burden.

It has not yet been proven that the immediate treatment of patients after the diagnosis of FL delays the incidence of transformation. No significant differences were observed in the cumulative risk of transformation between the WW cohort and immediate treatment cohort in the present study. A prospective observational study that mainly evaluated the incidence of histological transformation of FL has been conducted; 631 patients were enrolled, the transformation rate at 5 years was the highest in patients who were initially observed without any treatments, and immunochemotherapy improved post-transformation prognoses [11]. This observational study did not show definitive criteria for starting treatments or how to follow-up patients. A prospective interventional trial needs to be conducted in order to clarify whether the WW strategy affects the incidence of transformation of FL.

It was also essential for us to identify the best candidates for the WW strategy. In clinical practice, physicians typically take account of the tumor burden, which is based on the criteria advocated by Group d’ Etude des Lymphomes Folliculaires [4, 16], British National Lymphoma Investigation [5], and German Low-grade Lymphoma Study Group [17]. Rituximab plus chemotherapy is regarded as the standard treatment strategy for patients with HTB. On the other hand, it is acceptable to observe patients with LTB without any treatment until progression to HTB [18]. Although the WW cohort in the present study had more patients with LTB, no significant differences were observed in outcomes between the WW cohort and immediate treatment cohort. This result suggests that the WW strategy based on the tumor burden is acceptable in the rituximab era.

There were several limitations to this study. The number of events may not have been sufficient for evaluation in the analysis of OS and transformation rate. There might be various reasons why the risk of transformation was lower than we had expected: short duration of follow-up, difficulty in detecting the data on incidence of clinical transformation, and small number of the patients with high FLIPI2 score. Furthermore, the results may have been affected by the physicians’ choices in assigning patients to a cohort. In addition, the restrictions associated with a retrospective study added further obstacles.

In conclusion, the WW strategy did not have a negative impact on TTF, OS, or transformation in selected patients with FL. These results suggest that this strategy is still an acceptable approach for FL patients in the rituximab era. Further studies, particularly a prospective cohort study including an evaluation of optimal criteria for starting anti-lymphoma treatments, will confirm these results.
